# The “Bone Block Technique”: Reconstruction of Bone Defects Caused by Osteomyelitis Using Corticocancellous Bone Blocks from the Iliac Crest and the Induced Membrane Technique

**DOI:** 10.3390/life15091340

**Published:** 2025-08-25

**Authors:** Marc Hückstädt, Christian Fischer, Alexander Weissmann, Steffen Langwald, Patrick Schröter, Friederike Klauke, Thomas Mendel, Gunther O. Hofmann, Philipp Kobbe, Sandra Schipper

**Affiliations:** 1 Department of Trauma and Reconstructive Surgery, BG Klinikum Bergmannstrost Halle gGmbH, Merseburger Strasse 165, 06120 Halle (Saale), Germany; 2 Department of Trauma, Hand and Reconstructive Surgery, Friedrich-Schiller-University Jena, University Hospital Jena, Am Klinikum 1, 07747 Jena, Germany; 3 Department of Trauma, Hand and Reconstructive Surgery, Martin Luther University Halle-Wittenberg, University Hospital Halle, Ernst-Grube-Straße 40, 06120 Halle (Saale), Germany

**Keywords:** Bone Block Technique, segmental bone defect, corticocancellous bone graft, press-fit fixation, internal osteosynthesis, absolute stability, induced membrane technique, diamond concept, biological reconstruction

## Abstract

**Background:** The Induced Membrane Technique (IMT), commonly known as the Masquelet Technique (MT), has shown promising results in the reconstruction of bone defects caused by osteomyelitis. However, it is not a standardized surgical protocol but a treatment concept that has undergone various modifications, often yielding heterogeneous outcomes. **Methods:** This retrospective, single-center clinical cohort study included 49 patients treated with the Bone Block Technique (BBT) between 2013 and 2019 for bone defects resulting from osteomyelitis. The primary outcomes were time to bone healing, reinfection rate, and time to full weight-bearing. Additionally, infectious disease parameters, surgical site complications (SSCs), and epidemiological data were evaluated. **Results:** Data from 49 patients (mean age: 51 years, range: 17.6–76.9; 28.6% female) were analyzed, with a mean follow-up of 6.1 years (range: 4–10.5). The average bone defect length was 4.2 cm (range: 2.1–8.4 cm), predominantly involving the lower extremity. Primary bone consolidation was achieved in 93%, and secondary consolidation (requiring additional surgery) in 7%. Revision surgery due to recurrent infection was necessary in 16.6% of cases. The average time to full weight-bearing was 101.3 days. **Conclusions:** The BBT, as a modified approach based on the original IMT, represents a viable and reproducible option for bone defect reconstruction. When applied in accordance with the principles of the Diamond Concept, this technique facilitates reliable primary consolidation with a low complication rate.

## 1. Introduction

The reconstruction of large bone defects remains a significant challenge in musculoskeletal surgery. Distraction osteogenesis (DO) is currently considered the gold standard but is associated with high complication rates and prolonged treatment periods [1,2]. Frequent complications include recurrent infections, pin tract infections or loosening, axial deformities, peri-implant fractures, and insufficient consolidation or docking, all of which demand a high level of patient compliance [1,2,3,4]. Alternative methods such as vascularized fibula transfer are less established and not directly comparable to DO [5,6,7].

The Induced Membrane Technique (IMT) offers a cost-effective, reliable, and reproducible treatment option [8,9]. Originally introduced by Masquelet et al., the IMT is a two-stage procedure involving the temporary placement of a polymethylmethacrylate spacer to induce a vascularized membrane, followed by defect filling with cancellous bone grafts [10]. While the biological activity of the membrane contributes to graft integration, the technique provides limited initial structural stability and requires meticulous timing between stages. Numerous modifications have been introduced, resulting in heterogeneous outcomes. Variations in mechanical stabilization (internal vs. external fixation) and graft composition (autologous, allogenic, xenogenic) are ongoing subjects of discussion [6,7,9,11,12]. However, a standardized approach remains lacking [11,13,14].

This study presents a modified approach to the IMT, the Bone Block Technique (BBT). It represents a structurally focused, biologically driven alternative and involves the use of corticocancellous bone blocks, typically in combination with stable internal fixation, allowing for immediate mechanical integrity and one-stage reconstruction in selected cases. Unlike IMT and DO, the Bone Block Technique (BBT) does not rely on the formation of a biologically active membrane or prolonged bone transport, but instead emphasizes the mechanical and osteoconductive properties of the graft material itself. It strictly adheres to the principles of the Diamond Concept while simultaneously addressing the three primary causes of IMT failure: infectious, mechanical, and biological factors [5,15]. No biological adjuncts, growth factors, or BMP/PRP were used in any of the cases.

### 1.1. Surgical Technique

#### 1.1.1. BBT Stage I

**Figure 1:**
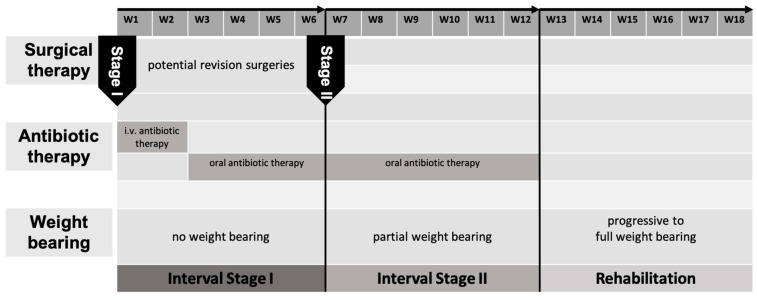
Hospital intern therapy regimen displaying the timeline of stage one and two of BBT.

In the early application of the IMT, revision surgeries were performed until no further microbiological or histopathological pathogens could be identified. At that point, antibiotic therapy was discontinued and only resumed in Stage II if pathogens were detected again. In recent years, we have transitioned to a strategy of minimizing revision surgeries, maintaining continuous antibiotic therapy up to and beyond Stage II of the IMT (Figure 1).

#### 1.1.2. BBT Stage II

Six weeks after Stage I, the reconstructive procedure (Stage II) is performed. The polymethyl methacrylate (PMMA) spacer is removed, bone resection margins are refreshed, avital bone is resected, and temporary osteosynthesis is replaced by definitive fixation if this has not already been done. Corticocancellous bone grafts (CCBGs) and cancellous bone (CB) are harvested from the anterior or posterior iliac crest, either ipsilaterally or contralaterally.

**Figure 2:**
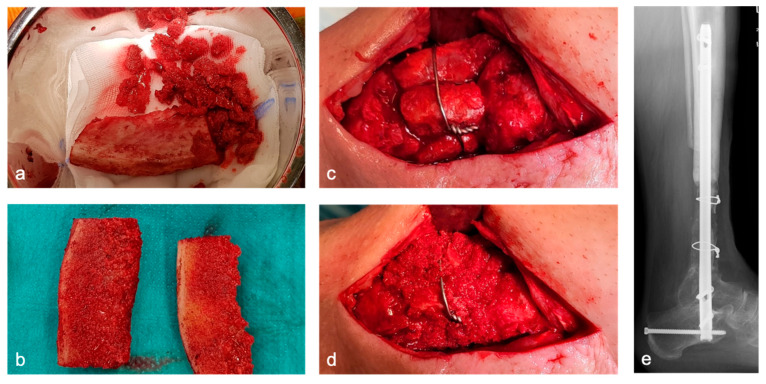
CCB and CB harvested from the iliac crest (**a**); division of a tricortical graft into two bicortical grafts (**b**); press-fit graft insertion with CB filling and cerclage fixation (**c**,**d**); postoperative radiograph (**e**).

Depending on the size of the defect, one or more CCBGs are harvested from the iliac crest (Figure 2a). Protective plating at the donor site is not performed in our approach. Tricortical grafts are divided into two bicortical grafts when appropriate, depending on the morphology and location of the defect (Figure 2b). The grafts are inserted into the defect using a press-fit technique and secured with screws or cerclage wires if necessary to enhance primary stability (Figure 2c–e). When intramedullary nailing is performed, bicortical grafts are placed circumferentially around the nail (Figure 2c). Remaining CB is packed into the defect and along the graft–host interface (Figure 2d). For defects > 5 cm, a single graft is typically insufficient. In such cases, multiple bone blocks are harvested and inserted either in parallel or in series (Figure 2c–e).

Internal osteosynthesis techniques were used in the majority of cases of biological bone-defect reconstruction. We adhere to the AO principles of fracture management, with particular emphasis on achieving absolute stability to promote primary bone healing via direct osteonal remodeling without callus formation [16,17,18,19]. In the epi- and metaphyseal regions, double locking plating was used, whereas diaphyseal defects were managed with a combination of intramedullary nailing and locking plate osteosynthesis. This approach enables stable osteosynthetic fixation with minimal interfragmentary strain (<0.15 mm gap), establishing the optimal mechanical environment for primary healing as defined by Perren’s strain theory, and also represents a cornerstone of the Diamond Concept in bone regeneration [20,21].

## 2. Materials and Methods

### 2.1. Study Design

This single-center, retrospective cohort study was conducted following approval by the responsible institutional ethics committee and in accordance with the Declaration of Helsinki (approval no. 86/21).

### 2.2. Data Acquisition

Retrospective data were collected for the period from May 2013 to December 2019. Patients older than 16 years with acute or chronic osteomyelitis and a post-debridement bone defect > 2 cm following Stage I surgery were included. Diagnosis of osteomyelitis was based on microbiological and histopathological findings. Patients with bone defects caused by trauma, non-union, or tumors were excluded. Patient identification and data collection were conducted using the hospital information system (HIS). Clinical and radiological follow-up was performed in our outpatient clinic at regular intervals.

### 2.3. Investigated Parameters

Collected data included epidemiological variables (gender, age, weight, BMI, ASA classification) and relevant comorbidities. Hospitalization time was defined as the inpatient duration following Stage II surgery. The bone defect size was measured intraoperatively. Details regarding autologous grafts (bi- or tricortical), orientation (parallel vs. serial), and fixation method (press-fit, cerclage, screw) were documented. The type of osteosynthesis (internal vs. external) was recorded.

Primary outcome measures were the rate and timing of osseous consolidation, reinfection rate, and time to full weight-bearing. Assessment of bone consolidation was based on CT imaging and independently performed by two trauma surgeons and one radiologist to enhance interobserver reliability and categorized as follows:

Primary: ≥50% cortical bridging proximally and distally without additional surgery;

Partial: <50% cortical bridging proximally and distally;

Secondary: Consolidation achieved after additional surgical intervention (e.g., further bone grafting, re-osteosynthesis).

Additional parameters included number of surgeries between Stages I and II, microbiological findings, recurrence of infection (defined as the need for surgical revision), and SSCs (e.g., hematoma, impaired soft tissue healing, neurovascular injury).

### 2.4. Statistical Analysis

Patient data were extracted and compiled in Microsoft Excel before statistical analysis using IBM SPSS Statistics (Version 26, Armonk, NY, USA). Descriptive statistics were used for all variables. Continuous data are presented as means ± standard error of the mean (SEM). Outliers (>2 standard deviations from the mean) were excluded. A *p*-value < 0.05 was considered statistically significant. Normality was tested, and parametric comparisons were conducted using the independent samples *t*-test when appropriate.

## 3. Results

**Figure 3:**
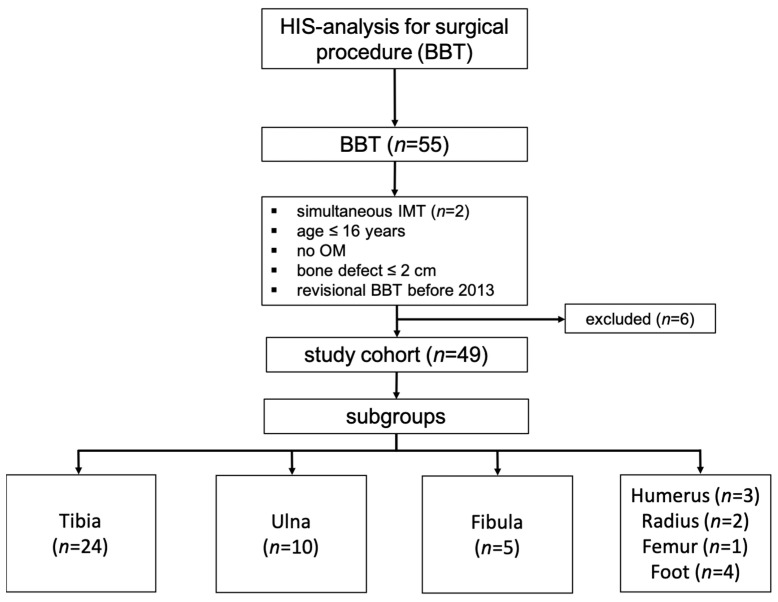
Study cohort and subgroup formation based on available hospital information system (HIS) data.

Between May 2013 and December 2019, 53 patients (55 cases) were treated using the Bone Block Technique (BBT). In two cases, surgery was performed at two anatomical sites (radioulnar and tibiofibular). For consistency, only the data from the larger defect site were included in the analysis. Patients who underwent BBT revisions before 2013 or whose bone defects were not caused by osteomyelitis were excluded (*n* = 6), resulting in a final study population of 49 patients. The majority of procedures were performed on the lower extremity (Figure 3).

### 3.1. Study Cohort

The mean patient age was 51 years (range: 17.6–76.9), with 14 females (28.6%) and 35 males (71.4%). The mean body mass index (BMI) was 29.2 (range: 19.5–48.7). Average follow-up was 6.1 years (range: 4–10.5). Relevant comorbidities included smoking (51%) and diabetes mellitus (18.4%); vascular diseases were not documented.

Fractures were classified as open or closed, with 38.8% classified as open fractures Type I/II according to the Tscherne and Oestern classification (Table 1) [22].

### 3.2. Surgical Data

The mean defect length was 4.2 cm (range: 2.1–8.4 cm). During Stage I, temporary internal osteosynthesis was used in 61.2% of cases, external fixation in 8.2%, and immobilization via casting in 30.6%. The non-weight-bearing period between Stage I and Stage II was 57.1 ± 4 days (range: 2–136 days).

In Stage II, 65.3% of patients received locking plate fixation, 30.7% intramedullary nailing, and 2% each received either external fixation or casting. Grafts were predominantly harvested from the anterior iliac crest (96%), with 80% being tricortical and 20% bicortical. In cases requiring multiple bone blocks, grafts were placed in parallel in 45% and in series in 8%.

The press-fit technique was used for graft insertion in 71% of patients; in the remaining 29%, additional fixation with screws or cerclage wires was necessary. Flap coverage for soft tissue defects was performed in 17 patients (34.7%) (Table 2).

### 3.3. Consolidation

Of the 49 patients, one was lost to follow-up, leaving 48 for consolidation assessment. Primary bone healing was achieved in 41 patients (93.2%). Three patients (6.1%) each demonstrated partial or secondary consolidation. All patients were permitted partial weight-bearing following Stage II. Full weight-bearing was achieved after an average of 101.3 days.

### 3.4. Infectiology

The most common pathogens were coagulase-negative staphylococci, Staphylococcus aureus, and mixed infections. On average, patients underwent 3.3 surgeries between Stages I and II, with a mean interval of 57.1 days. Eight patients (16.6%) required revision surgery due to reinfection. In 75% of these cases, the infecting pathogen changed; in 25%, it remained the same. There was no correlation between the number of surgeries and rates of bone healing or reinfection.

In 12 patients, pathogens were still detectable during Stage II; however, this did not adversely affect bone healing outcomes.

Over the study period, the Stage I treatment protocol evolved. Initially, antibiotic therapy was discontinued once pathogens were no longer detectable before Stage II (19 patients, 38.8%). Later, continuous antibiotic therapy through and beyond Stage II was implemented (30 patients, 61.2%). Recurrent infections occurred in four cases in each of the two subgroups (Table 3). There was no significant difference in the recurrence rate between the subgroup with an antibiotic-free interval before Stage II and the subgroup receiving continuous antibiotic therapy before and after Stage II (*p* = 0.694). Thirty-two patients received a 6-week course of antibiotics after Stage II, whereas 17 patients received no antibiotic therapy after Stage II. Recurrent infections likewise occurred in four cases in each of these subgroups, again without a significant difference (*p* = 0.423). Due to the low number of recurrent infections, no further distinction was made between superficial and deep infections.

### 3.5. Complications

Surgical site complications (SSCs) such as impaired wound healing or hematoma occurred in four patients (8%) following Stage II. Refractures occurred in three patients (6%) after implant removal, with one requiring revision osteosynthesis. No amputations were necessary. Donor site morbidity was minimal; only one patient developed a temporary lateral femoral cutaneous nerve injury.

## 4. Discussion

This study contributes to the growing body of literature on the Induced Membrane Technique (IMT) and its modifications, particularly for the treatment of post-infectious bone defects. However, direct comparisons with other studies are limited due to considerable methodological heterogeneity, including differences in patient demographics, defect etiology, defect size and location, follow-up duration, fixation strategies, and grafting techniques [11,23].

In our cohort, the mean defect length was 4.2 cm—slightly smaller than the averages reported in recent reviews by Morelli (5.53 cm) and Mi (6.32 cm) [11,23]. Importantly, all cases in our study were related to secondary osteomyelitis, limiting direct comparability with studies that include only acute septic defects [21,23,24]. The average follow-up of 6.2 years in our cohort is significantly longer than the 12-month average in most reviews, supporting the validity of our long-term results [11,23].

### 4.1. Fixation Strategies in Stage I and II

Fixation strategies for Stage I vary widely in the literature. In our series, internal fixation with locking plates or intramedullary nails was used predominantly, with fewer cases utilizing external fixation. No consensus exists regarding the optimal fixation method in the presence of infection—some authors prefer internal fixation, others external [1,6,9,21,24].

Similarly, in Stage II, our preference is for internal fixation (locking plates: 65.3%, nails: 30.6%), consistent with other reports [25,26]. We emphasize that stable mechanical conditions are critical to success: the press-fit insertion of corticocancellous grafts must be combined with absolute fixation stability. To promote enchondral ossification, bone–graft interfaces must allow <0.2 mm micromotion and <2% strain [27,28]. Split bi- or tricortical iliac crest grafts were additionally stabilized using cerclage wiring or screw osteosynthesis.

Although biodegradable polymer-based spacers have been proposed as a potential alternative to polymethylmethacrylate (PMMA) in two-stage bone reconstruction procedures—particularly with the aim of avoiding a second surgical stage and minimizing spacer-related complications—their use remains experimental and is not yet supported by robust clinical data. PMMA spacers, by contrast, offer proven advantages in terms of mechanical stability, space maintenance, and the possibility of targeted local antibiotic delivery, which is especially relevant in the setting of post-infectious bone defects. Furthermore, the bioactivity of the induced membrane formed around PMMA has been well characterized, whereas comparable data for biodegradable materials are limited. Recent experimental studies using biodegradable scaffolds such as polycaprolactone fumarate or PLGA-based composites have shown promising results in preclinical models; however, these materials currently lack the structural integrity and long-term clinical validation required for routine clinical use. For these reasons, PMMA remains the standard of care in Induced Membrane Techniques, particularly in complex cases requiring reliable mechanical and anti-infective properties [11,23].

### 4.2. Grafting Technique and Bone Healing

A key innovation in our BBT protocol is the use of load-bearing bi- and tricortical grafts from the iliac crest, which offer biomechanical advantages over purely cancellous grafts. With this technique, we achieved a 93% primary consolidation rate, comparable to results from Morelli (89.7%) and Mi (92.4%) [11,23]. The added structural stability allowed for immediate partial weight-bearing (up to 20 kg), helping to avoid complications associated with prolonged immobilization.

Full weight-bearing was achieved after a mean of 15 weeks—earlier than reported in other studies [12,21,25,29]. This earlier mobilization likely contributed to improved functional outcomes and faster reintegration into daily activities.

**Figure 4:**
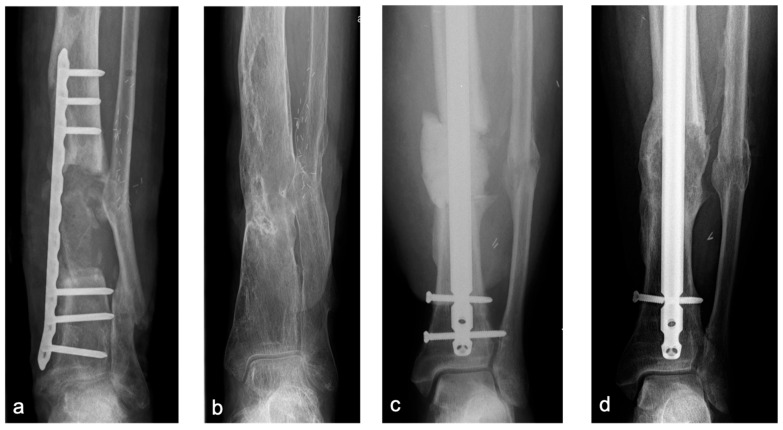
Case 1 (**a**,**b**): Bone defect of the distal tibia (7 cm), reconstruction using the BBT (**a**): postoperative X-ray, (**b**): at 10-year follow-up. Case 2 (**c**,**d**): Bone defect of the distal tibia (4 cm), Step I of the BBT (**c**): postoperative X-ray with temporary intramedullary nail osteosynthesis and PMMA spacer; (**d**): at 4-year follow-up after Step II of the BBT.

**Figure 5:**
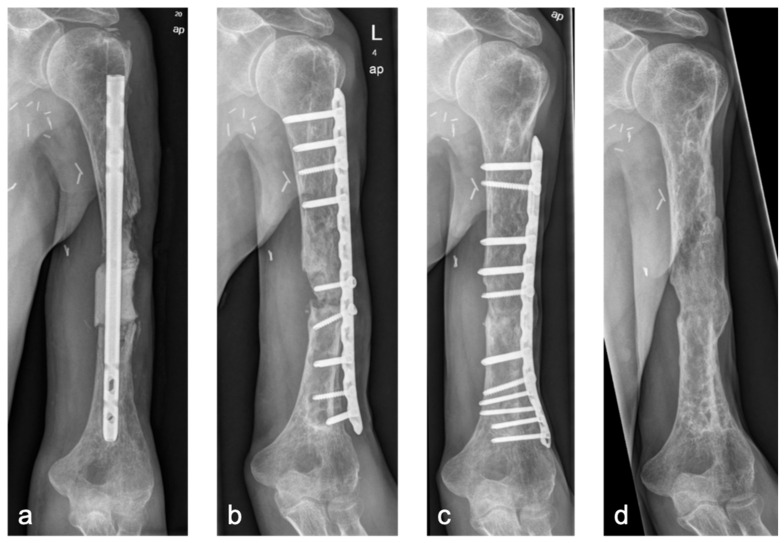
Case 3 (**a**–**d**): Bone defect of a humeral diaphysis (4 cm) (**a**): Temporary intramedullary nail with PMMA spacer, (**b**): Distal non-union and mechanical failure of the BBT 1 year after Step II, (**c**): Secondary consolidation 1 year after revision BBT with additional corcticocancellous bone grafting, (**d**): Remodeling 3 years after revision BBT and implant removal.

In the lower extremity, long-term follow-up demonstrated robust remodeling (Figure 4). However, in the upper extremity, particularly the humerus, two of three cases developed aseptic non-unions. In both, single-plate fixation was used (Figure 5). Given the mechanical demands of the upper limb—characterized by long lever arms and relatively low axial loading—achieving absolute stability and primary bone healing through compression remains challenging. Therefore, we strongly recommend angular stable double-plate fixation to ensure sufficient stability and reduce the risk of fixation failure, in line with current biomechanical and clinical recommendations [5,30,31].

### 4.3. Recurrent Infections

The overall reinfection rate of 16.6% in our study—over a follow-up of 73.2 months—appears low, especially given that some studies report higher rates (16–59%) over shorter follow-up periods (12–13 months) [22,30]. The reduced reinfection rate may be attributed to the choice of internal fixation, reduced surgical burden between stages, and refined antibiotic strategies [31,32,33].

Pathogen persistence at Stage II did not correlate with poorer outcomes, possibly reflecting the protective effect of the induced membrane [21,24,27]. Patients requiring flap reconstruction had a higher, though not statistically significant, rate of reinfection (23.5% vs. 12.5%). Complex soft tissue injuries remain a known risk factor for infection [28].

A six-week postoperative antibiotic course appeared to reduce recurrence rates, supporting the importance of tailored antimicrobial therapy in high-risk or persistent infections [8].

### 4.4. Complications

Minor surgical site complications (hematoma, impaired healing) occurred in 8% of cases. Major complications such as thrombosis or amputation were not observed. Refractures after implant removal occurred in 6%, in line with published reports [5]. We recommend retaining implants for at least 18 months post healing to prevent such events.

### 4.5. Limitations

The study is limited by its retrospective, non-randomized design and the absence of a control group (original Masquelet technique/IMT, distraction osteogenesis/Ilizarov method). Additionally, the sample size is relatively small. Therefore, the findings should be interpreted with caution. Future prospective studies with larger cohorts and control arms (e.g., distraction osteogenesis or free fibular grafting) are needed to validate the BBT.

### 4.6. Future Directions

Further exploration of new materials such as bioactive scaffolds or 3D-printed implants may expand future treatment options.

Bioglass exhibits excellent bioactivity and osteoconductive properties, forming a hydroxy-carbonate apatite (HCA) layer upon contact with physiological fluids, thereby enhancing bone bonding and stimulating mineralization. It promotes osteoblast activity and accelerates osteogenesis, particularly when used in mesoporous scaffold forms that increase surface area and osteogenic gene expression. Additionally, bioglass demonstrates notable antimicrobial and angiogenic effects; formulations such as S53P4 show broad-spectrum antibacterial activity via local pH elevation and ion release, with clinical success rates near 90% in treating chronic osteomyelitis. These glasses also support neovascularization, critical for healing regarding large bone defects [34,35,36].

However, mechanical and degradation limitations persist. Bioglass is inherently brittle, with low fracture toughness and tensile strength, rendering it unsuitable for load-bearing applications. Sintering commercial variants like 45S5 into mechanically stable scaffolds remains challenging due to crystallization. Its typically slow degradation may not align with the rate of bone regeneration, and the biological impact of sustained ion release warrants further investigation. Lastly, clinical evidence remains heterogeneous, with modest benefits reported in periodontal applications and outcomes varying by formulation, particle size, and defect site, underscoring the need for high-quality randomized controlled trials [37,38].

Recent advances in biomaterials and implant design have enabled personalized, load-bearing solutions. Custom 3D-printed titanium lattice implants (75% porosity, 300–900 µm pores) show excellent long-term osseointegration, mechanical stability, and functional outcomes in large bone defects. Similarly, combining porous titanium prostheses with the Masquelet technique yields favorable recovery and structural restoration in complex femoral reconstructions [39,40,41].

Next-generation bioceramic and polymer composites, including resorbable tricalcium phosphate bioinks and hydroxyapatite-coated PEEK/PEKK implants, support early vascularization and osteogenic differentiation while maintaining mechanical strength.

Platform innovations focus on hierarchical porosity, biofunctionalization, and smart multifunctional implants. AI-assisted design is accelerating custom implant development from imaging data.

Clinical translation is progressing, with trials evaluating 3D-printed titanium in long bone reconstruction and PEEK or bioceramic scaffolds in cranial applications, demonstrating early safety and efficacy, though long-term data remain limited [42,43].

Despite these promising modern approaches to bone defect reconstruction—such as bioglass and 3D-printed implants—these techniques remain costly and not universally accessible. In our experience, biological reconstruction has proven to be a practical, cost-effective, and reproducible alternative. Even large, critical-sized bone defects exceeding 10 cm can be successfully reconstructed using a combination of autologous and allogeneic grafting within the Induced Membrane Technique (IMT), known as the “Pearl-String Technique” [44,45].

## 5. Conclusions

Mid-term outcomes of the Bone Block Technique (BBT) are encouraging, demonstrating its efficacy in the reconstruction of medium to large bone defects following osteomyelitis. The high rate of bone consolidation and the comparatively low reinfection rate highlight the potential of this approach, particularly when using structurally stable corticocancellous bone grafts in combination with internal fixation. While the development of a standardized protocol remains challenging due to the complexity and variability inherent to bone defect reconstruction, both Stage I and Stage II offer a structured approach that can support surgical planning. Nonetheless, further reduction in infection recurrence remains essential to enhance the reproducibility and long-term reliability of the technique.

## Figures and Tables

**Table 1:** Demographics and clinical characteristics of the study cohort.

Gender	Age [years]	BMI [KG/m^2^]	Smoking	Diabetes	ASA Classification	Initial Open Fractures
male: 35 (71.4%) female:14 (28.6%)	51.45 y ± 2.09 y (17.6–76.85)	29.23 ± 0.96 (19.45–48.67)	*n* = 25 (51.0%)	*n* = 9 (18.4%)	ASA 1: *n* = 10 (20.4%) ASA 2: *n* = 35 (71.4%) ASA 3: *n* = 4 (8.2%)	*n* = 19 (38.8%)

**Table 2:** Operative data: fixation methods, defect dimensions, grafting technique, and flap coverage.

Stage Two Fixation	Osteosynthese und MT Simultan	Bone Defect Length [mm]	Amount of Iliac Crest Grafts Used	Type of Iliac Crest Grafts Used	Origin of Iliac Crest Grafts	Arrangement of Iliac Crest Grafts	Fixation of Iliac Crest Grafts	Flap Coverage
locking plate *n* = 32 (65.3%) intramedullary nail *n* = 15 (30.7%) external fixation (Ilizarov) *n* = 1 (2%) cast *n* = 1 (2%)	*n* = 20 (40.8%)	42.08 ± 2.19 mm (21.3–83.9)	one graft *n* = 27 (55.1%) two grafts *n* = 21 (42.9%) three grafts *n* = 1 (2%)	bicortical *n* = 10 (20.4%) tricortical *n* = 39 (79.6%)	anterior *n* = 47 posterior *n* = 1 anterior + posterior *n* = 1	single *n* = 23 (46.9%) parallel *n* = 22 (44.9%) In line *n* = 4 (8.2%)	Press-fit *n* = 35 (71.4%) cerclage wire *n* = 2 (4.1%) screws *n* = 12 (24.5%)	*n* = 17 (34.7%)

**Table 3:** Perioperative infectiology and antibiotic treatment in Stage I.

Pathogens Detected at Stage One BBT	Recurrent Infection (*n* = 8)	Revision Surgeries Before Stage Two BBT	Interval Between Stage One and Two BBT [Days]	Temporary Fixation After Stage One BBT	Pathogen Detection at Stage Two BBT	Patients Without Antibiotic Therapy Before Stage Two BBT	Patients with Continuation of Antibiotic Therapy After Stage Two BBT
coagulase-negative staphylococcus *n* = 28 staphylococcus aureus *n* = 16 mixed infection *n* = 18 enterococcus *n* = 4 streptococcus *n* = 3 cutibacterium acnes *n* = 10 Gram-negative bacteria *n* = 6 others *n* = 8	coagulase-negative staphylococcus *n* = 4 staphylococcus aureus *n* = 3 enterococcus *n* = 1	*n*= 3.3 ± 2.1 (1–8)	57.1 ± 4d (2–136)	locking plate *n* = 19 (38.8%) intramedullary nail *n* = 6 (12.2%) carbon rod *n* = 1 (2%) external fixateur *n* = 4 (8.2%) K-wire and cast *n* = 4 (8.2%) cast *n* = 15 (30.6%)	*n* = 12 (24.5%)	*n* = 19 (38.8%)	*n* = 32 (65.3%)

## Data Availability

The data presented in this study are available on request from the corresponding author, due to (specify the reason for the restriction) restrictions related to privacy and data protection regulations.

## Abbreviations

The following abbreviations are used in this manuscript:

BBT	Bone block technique
IMT	Induced membrane technique
MT	Masquelet technique
SSC	Surgical site complication
DO	Distraction osteogenesis
PMMA	Polymethylmethacrylate
CCBG	Corticocancellous bone graft
CB	Cancellous bone
HIS	Hospital information system
FU	Follow-up
SEM	Standard error of the mean
BMI	Body mass index
ASA	American Society of Anesthesiologists
cm	Centimeters
